# Stereopsis deficits in patients with schizophrenia in a Han Chinese population

**DOI:** 10.1038/srep45988

**Published:** 2017-04-12

**Authors:** Li Hui, Hai Sen Xia, An Shu Tang, Yi Feng Zhou, Guang Zhong Yin, Xing Long Hu, Xiang Dong Du, Yong Tang

**Affiliations:** 1 Wenzhou Kangning Hospital, Wenzhou Medical University, Wenzhou, Zhejiang, PR China; 2 Institute of Mental Health, Suzhou Psychiatric Hospital, The Affiliated Guangji Hospital of Soochow University, Suzhou, Jiangsu, PR China; 3 Mental Health Center of Anhui Province, Hefei, Anhui, PR China; 4 Vision Research Laboratory, School of Life Science, University of Science and Technology of China, Hefei, Anhui, PR China

## Abstract

Although cognitive and sensory deficits have been identified as a core feature of schizophrenia, only a small portion of visual sensorium has been explored. To date, studies on visual system of three-dimensional percepts based on two-dimensional information still are limited. This study is the first to examine the integrity of stereopsis of schizophrenia in a Han Chinese population, and to further investigate the correlation of stereopsis with clinical symptoms. 100 patients with schizophrenia and 80 healthy controls were recruited. We assessed stereoacuity using the Titmus Stereopsis Test and clinical symptoms using Chinese versions of the Scales for the Assessment of Positive and Negative Symptoms (SAPS and SANS). There was a significant difference in log seconds of arc between two groups (p < 0.0001). The percentage of patients with correct stereopsis detection was significantly reduced at 400, 200, 140, 100, 80, 60, 50, and 40 seconds of arc than healthy controls (all, p < 0.01). Log seconds of arc in patients was not correlated with total scores and subscores of SAPS and SANS (all, p > 0.05). Our findings support that patients with schizophrenia have a marked deficit of stereopsis in a Han Chinese population. However, clinical symptoms do not influence stereopsis of schizophrenia.

Cognitive and sensory deficits have been identified as a core feature of psychiatric disorders, especially in patients with schizophrenia^1^,^2^,^3^. Visual perception could be the result of complex mechanisms of integration of visual information that could come from magnocellular and/or parvocellular visual pathways, or cortical integration of sensory information with top-down signals^4^,^5^. Previous studies have indicated that patients with schizophrenia experience greater deficits of early visual processing and integrative visual processes compared with healthy controls^6^,^7^. Another study has found that numerous visual and perceptual deficits appear in patients with schizophrenia, their first-degree relatives and schizotypal patients^8^. Moreover, visual deficits have been reported before onset of schizophrenia^9^,^10^,^11^,^12^. The deficits of visual perception could further cause high morbidity, poor quality of life, even unemployment for schizophrenia. Therefore, the deficits of visual perception should become a prodromal symptom and therapeutic target for schizophrenia, and the underlying mechanism of visual and perceptual deficits in patients with schizophrenia should be further investigated.

A fundamental component of early visual processing is stereopsis that which literally means “solid vision”. It emerges as a result of the disparity between left- and right eye images due to their horizontal displacement (~60 mm)^13^. When two images are fused in cerebral cortex, the disparity further gives rise to a three-dimensional image. A critical function of early visual system should be the generation of three-dimensional percepts based on two-dimensional information. Although some studies have focused on two-dimensional visual processing in patients with schizophrenia, studies on three-dimensional percepts still are limited. To date, only two studies on patients with schizophrenia directly assess stereopsis illusion using tests of stereoacuity that are commonly used in routine visual examination^5^,^14^. It has been found that stereopsis in patients with schizophrenia is impaired, and clinical psychiatric symptoms are not correlated with stereopsis in patients with schizophrenia using the Graded Circle test that is derived from the Wirt stereo^5^,^15^. Schizophrenic and schizoaffective patients experience greater stereopsis deficits than healthy controls, and there are no associations between clinical psychiatric symptoms and stereopsis in patients with schizophrenia using the Graded Circle test along with the random dot stereograms and Frisby Stereo Test^14^,^16^,^17^. These findings suggested that there could be a significant difference in stereopsis between patients with schizophrenia and healthy controls in a Han Chinese population. However, stereopsis of Han Chinese schizophrenia still did not been investigated in previous studies. Moreover, racial difference has been found to be involved in cognitive function^18^,^19^. Therefore, this study is the first to recruit Han Chinese schizophrenia to examine the integrity of stereopsis, and to further investigate the correlation of stereopsis with clinical psychiatric symptoms in patients with schizophrenia. We hypothesized that: (a) patients with schizophrenia have poorer stereopsis than healthy controls in a Han Chinese population; (b) clinical psychiatric symptoms do not influence stereopsis in patients with schizophrenia.

## Results

Clinical and demographic characteristics were summarized in Table 1. Patients with schizophrenia and healthy controls did not significantly differ in gender (χ^2^ = 1.57, p = 0.69), age (t = −1.86, p = 0.06) and education (t = −0.19, p = 0.85). Mean and standard (mean ± SD) of age of illness onset, illness duration, SAPS and SANS scores in patients with schizophrenia were 25.7 ± 10.3 years, 16.7 ± 6.6 months, 14.8 ± 13.4 and 35.0 ± 20.4. Moreover, median of stereoacuity thresholds in patients with schizophrenia and healthy controls were 60 and 40 seconds of arc.

**Figure 1:**
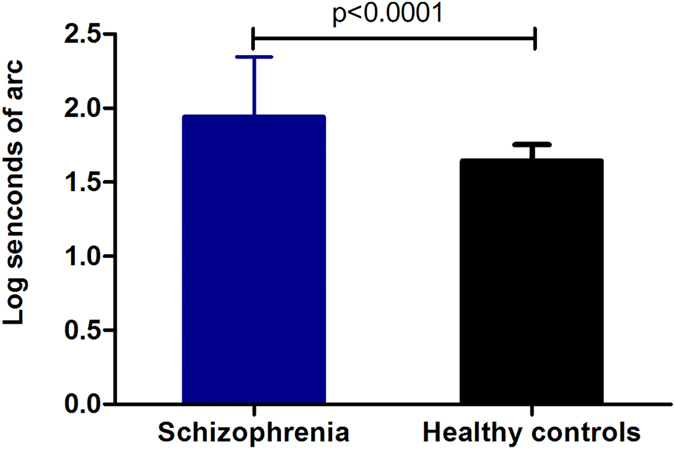
Difference in log seconds of arc between patients with schizophrenia and healthy controls (1.94 ± 0.41 vs 1.64 ± 0.11, t = 6.32, p < 0.0001).

Stereoacuity data were transformed for log seconds of arc in all subjects. The analysis of a two-tailed Student *t* test showed that log seconds of arc in patients with schizophrenia (mean = 1.94, median = 1.78, 95% confidence interval [CI]: 1.86–2.02) was significantly elevated compared with healthy controls (mean = 1.64, median = 1.60, 95% CI: 1.62–1.67, t = 6.32, df = 178, p = 0.000) (Fig. 1). Further analysis found that there were the significant differences in log seconds of arc between female patients and female healthy controls (t = 4.22, df = 97, p = 0.000), and between male patients and male healthy controls (t = 4.67, df = 79, p = 0.000). Moreover, there were no differences in log seconds of arc between female and male patients (t = 1.24, df = 98, p = 0.22), and between female and male healthy controls (t = −0.24, df = 78, p = 0.82).

The percentage of patients with correct stereopsis detection was significantly reduced at 400 (z = 2.90, p = 0.004), 200 (z = 3.2, p = 0.001), 140 (z = 4.01, p = 0.000), 100 (z = 4.72, p = 0.000), 80 (z = 5.32, p = 0.000), 60 (z = 6.20, p = 0.000), 50 (z = 6.27, p = 0.000), and 40 (z = 5.71, p = 0.000) seconds of arc compared with healthy controls (Fig. 1). Further correlation analysis showed that log seconds of arc was not association with age, education, onset of age, duration of illness, total score and subscores of SAPS and SANS in patients with schizophrenia (all, p > 0.05).

## Discussion

To our knowledge, this study is the first to investigate the integrity of stereopsis in patients with schizophrenia in a Han Chinese population. We found that patients with schizophrenia had a marked deficit of stereopsis compared with healthy controls in a Han Chinese population. However, the effect of clinical psychiatric symptoms on stereopsis was not found in patients with schizophrenia.

Our finding showed that stereopsis in patients with schizophrenia was impaired in a Han Chinese population, which is consistent with our provided hypothesis that patients with schizophrenia have poorer stereopsis than healthy controls in a Han Chinese population. Two previous studies have shown that patients with schizophrenia experienced greater stereopsis deficits than healthy controls^5^,^14^. The generation of three-dimensional percepts has been reported to be governed by cerebral cortex, especially extrastriate cortex that abnormal activity of which has been found in patients with schizophrenia^20^,^21^,^22^, which could further lead to a marked deficit of stereopsis in patients with schizophrenia. Interestingly, a recent study has found that stereopsis deficits in patients with Parkinson were associated with gray volume reduction in the right extrastriate visual cortical, which could further implicate cortical visual dysfunction in patients with Parkinson^23^. Therefore, further studies on the association between stereopsis and extrastriate cortex in patients with schizophrenia should be performed in the future.

**Figure 2:**
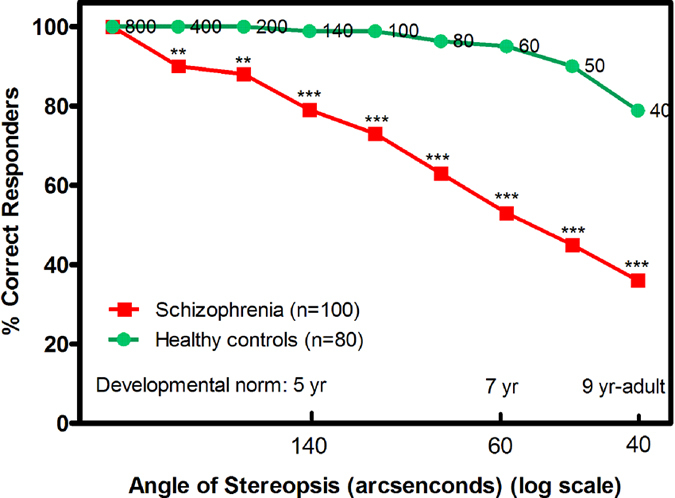
Percent of group performing correct stereopsis detection at indicated angles of stereopsis. Smaller angle of stereopsis correspond to increased task difficulty. **z = 2.9–3.2, p < 0.01. ***z = 4.01–6.27, p < 0.001.

We did not find the correlation of stereopsis with clinical psychiatric symptoms in patients with schizophrenia, which is line with our provided hypothesis that clinical psychiatric symptoms do not influence stereopsis in patients with schizophrenia. It has been found that steropsis was not significantly related to clinical psychiatric symptoms in patients with schizophrenia^5^,^14^. Moreover, our results were also accorded with schizophrenic neurodevelopmental theories. Normally, stereopsis in infancy began to development, and stereopsis in 9-year-old children reached in adult level^24^. Median stereopsis threshold observed in patients with schizophrenia in this study, 60 arcseconds (Fig. 2), was normally reached by age 7^24^,^25^, suggesting that stereopsis in patients with schizophrenia was impaired during childhood, and further indirectly supporting that clinical psychiatric symptoms were not associated with stereopsis in patients with schizophrenia. Therefore, such evidence suggested that stereopsis deficits should be regarded as a vital prodromal symptom for schizophrenia, which should be further developed a risk identification methodology of schizophrenia. Although a recent study showed that young subjects with clinical high risk of developing schizophrenia had normal stereopsis compared with healthy controls, the sample size was very small, and we also did not know that how many clinical high risk subjects would develop patients with schizophrenia^26^, which could lead to the possibility of false-positive findings. Thus, stereopsis of clinical high risk subjects should be further investigated to help predicting the risk of developing schizophrenia in longitudinal studies on a large sample.

Several limitations in this study should be noted. First, this study was case-control and cross-sectional, therefore the explanation of causal relationships was rather caution. Second, “aged 13–51 years” other than “18–35 years” was confirmed as inclusion criteria in this study, which could cause bias of diagnosis for schizophrenia in a very young age, such as 13. Thus, further study should be performed in schizophrenia between 18–35 years. Third, antipsychotic information was not collected, and the effect of antipsychotics on stereopsis of schizophrenia could not been further analyzed in this study. Thus, further study should collect antipsychotics information, and analyze the correlation of antipsychotics with stereopsis of schizophrenia. Forth, this study on schizophrenia under treatment could not exclude the effect of antipsychotics on stereopsis and clinical symptoms, which could lead to bias of our findings. Thus, further study on first-episode drug-free schizophrenia still need to be performed in the future. Fifth, other demographic and clinical information including smoking, age of first hospitalization, and hospitalization number were not collected, which should be considered in statistical analysis because they could influence stereopsis in patients with schizophrenia. Finally, population stratification of our sample could be confounders. However, all subjects in this study were Han Chinese population from Hefei area, which could not influence our findings.

In summary, we found that stereopsis in patients with schizophrenia was impaired in a Han Chinese population, and clinical psychiatric symptoms did not influence stereopsis in patients with schizophrenia. However, although our findings adapting a large sample (100 patients with schizophrenia and 80 healthy controls) were consistent with two previous studies^5^,^14^, the neural mechanisms of stereopsis deficits in patients with schizophrenia remained to be not fully known. Therefore, further studies should determine the neural mechanisms of stereopsis deficits in patients with schizophrenia, and whether stereopsis deficits could be regarded as a vital prodromal symptom for schizophrenia.

## Methods

### Ethics Statement

This study was carried out in accordance with the approved guidelines and regulations during the period between September 2013 and October 2015 in Hefei, China. The research protocol and informed consent were approved by the Institutional Review Board of Mental Health Center of Anhui Province; a Hefei City owned psychiatric hospital with 1530 beds. This hospital had a catchment area with a population of approximately 5,700,000. A clinical psychiatrist explained research protocol and procedures to potential subject. The description of this study was tailored to maximize understanding of the subjects using local language appropriate to the subject’s level of comprehension, and emotional readiness. If the subject was willing to consent to participate in this study, this psychiatrist would provide an in-depth description to the subject and their guardians. The subject must provide written informed consent to participate in this study.

### Subjects

Patients with schizophrenia (n = 100; male/female = 46/54) were recruited from the inpatient unit of Mental Health Center of Anhui Province. Inclusion criteria were: (a) aged 13–51 years; (b) Had been diagnosed with schizophrenia based on the Diagnostic and Statistical Manual of Mental Disorders, 4th edition (DSM–IV) [American Psychiatric Association, 1994]; (c) received education for at least 4 years; (d) no more than 2 years illness duration, and illness severity for mild using the assessment of the Clinical Global Impressions (CGI), (e) received a stable dose of oral antipsychotics for at least 3 months before entry into this study, and (f) provided written informed consent and were able to take part in stereopsis assessment. Diagnoses were made for each patient by two independent experienced psychiatrists and confirmed using the Structured Clinical Interview for DSM-IV.

Healthy controls (n = 80; male/female = 35/45) were recruited at the same time from the local Hefei area. Current mental status and personal or family history of mental disorders were assessed using unstructured interviews. None of healthy controls presented a personal or family history of psychiatric disorders.

All subjects were Han Chinese, and were in good physical health with no history of visual or ocular pathology. Any subjects with abnormalities were excluded including amblyopia, color-blindness, strabismus, glaucoma, diabetic retinopathy, schizoaffective disorders, dementia, neurodegenerative and neurological disorder, and cerebrovascular disease. Neither patients with schizophrenia nor healthy controls were experiencing drug or alcohol abuse/dependence.

### Clinical measures

A detailed questionnaire including gender, age, education, corrected vision, medical history, physical and psychotic examination was obtained from patients with schizophrenia and healthy controls. Additional information was collected from available medical records.

Two experienced psychiatrists (who were blind to the clinical status of patients with schizophrenia) assessed the positive and negative psychiatric symptoms using Chinese versions of the Scales for the Assessment of Positive and Negative Symptoms (SAPS and SANS)^27^,^28^. The SAPS and SANS consisted of 34 and 24 items, respectively, scored on six-point likert scales (from 0 = not present to 5 = severe). The SAPS contained four subscales: hallucinations, delusions, bizarre behavior and positive formal thought disorder. The SANS was composed of five subscales: affective blunting, alogia (impoverished thinking), avolition/apathy, anhedonia/ascociality and attention. The attending psychiatrists were simultaneously trained in the use of SAPS and SANS before this study was initiated. The Chinese versions of two scales had good reliability and validity^29^ The SAPS and SANS reported in this current study are total scores.

**Figure 3:**
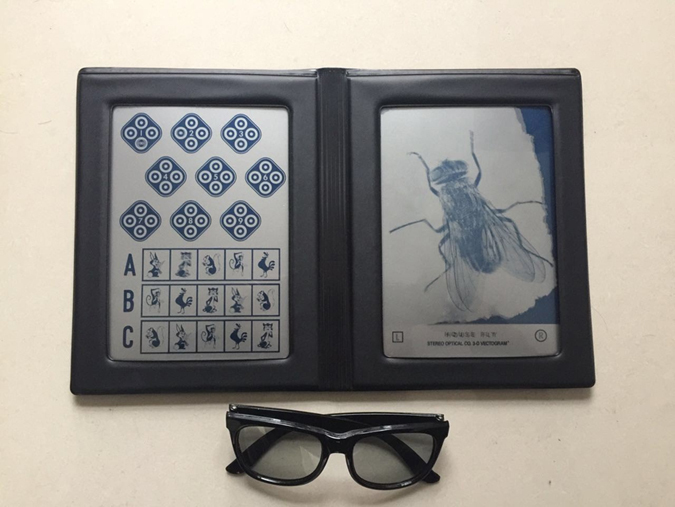
Titmus Stereopsis Test.

The stereoacuity under natural light was evaluated using the Titmus Stereopsis Test (Stereo Optical Co., Chicago, IL, USA) which consists of a large-disparity housefly, three series of animals, and nine sets of circles (Fig. 3). The housefly establishes the presence of gross stereopsis. The series of animals, from which a forward-appearing one is selected, are usually used for young children. The circle patterns provide a finely graded sequence for critical testing, and are designed mainly for adults. In each circle set there are 4 circles, and only one of them has a degree of crossed disparity, which makes that this circle seems to be closer to subjects than others. Subjects should indicate the closer circle within each set. With the decrease of the degree of crossed disparity, the difficulty level in stereopsis increases. A total of 9 levels, ranging from 40 to 800 arc seconds, are applied in circle test. Stereoacuity is recorded with the most difficult level reached by subjects. When testing, subjects viewed the images through polarizing spectacles with a distance of 40 cm. Also, the booklet was held perpendicular to the subject’s visual axis. The fly was shown first. If a positive response was given, circle test continued. Moreover, the eligible subject was accord with the following conditions of the stereoacuity assessment: (a) vision acuity of left and right eyes respectively reached at least 0.6 after correction, and (b) the acuity discrepancies between two eyes were no more than 1 line of E Standard Logarithm Eyesight Table.

### Statistical Analysis

Data analysis in this study was performed using Statistical Package for the Social Sciences (SPSS) Version 17.0 (SPSS Inc., Chicago, IL). The differences between patients with schizophrenia and healthy controls were compared using a two-tailed Student *t* test for age, education and log seconds of arc and χ^2^ test for gender. To analyze the correlation between log seconds of arc and age, education, age of illness onset, illness duration, clinical psychiatric symptoms in patients with schizophrenia, the Pearson’s correlation test was performed. Stereoacuity thresholds were defined as the minima angle of stereopsis at which the subject responded correctly. The proportion of each group responding correctly at each angle of stereopsis was compared using a z-transformed Mann-Whitney *U* test. All comparisons were two-sided with a significance level of 5%. Data were presented as mean ± SD. Figure was performed using GraphPad Prism Version 5.0 (GraphPad Software Inc., San Diego CA).

## Additional Information

**How to cite this article**: Hui, L. *et al*. Stereopsis deficits in patients with schizophrenia in a Han Chinese population. *Sci. Rep.* **7**, 45988; doi: 10.1038/srep45988 (2017).

**Publisher's note:** Springer Nature remains neutral with regard to jurisdictional claims in published maps and institutional affiliations.

## Figures and Tables

**Table 1:** Demographic and clinical characteristics of patients with schizophrenia (n = 100) and healthy controls (n = 80).

	Schizophrenia	Healthy controls	χ^2^ or t	df	P value
	n = 100	N = 80			
Gender (male/female)	46/54	34/45	1.57	1	0.69
Age (years)	27.1 ± 10.8	30.3 ± 12.3	−1.86	178	0.06
Education (years)	9.4 ± 2.7	9.5 ± 2.9	−0.19	178	0.85
Age of illness onset (years)	25.7 ± 10.3				
Duration of illness (months)	16.7 ± 6.6				
SAPS score	14.8 ± 13.4				
SANS score	35.0 ± 20.4				

Note: *P < 0.05. SAPS and SANS = the Scales for the Assessment of Positive and Negative Symptoms.
