# Erratum: Stereopsis deficits in patients with schizophrenia in a Han Chinese population

**DOI:** 10.1038/srep46812

**Published:** 2017-05-16

**Authors:** Li Hui, Hai Sen Xia, An Shu Tang, Yi Feng Zhou, Guang Zhong Yin, Xing Long Hu, Xiang Dong Du, Yong Tang

Scientific Reports 7: Article number: 4598810.1038/srep45988; published online: 04 12 2017; updated: 05 16 2017

In this Article, Yi Feng Zhou is incorrectly listed as being affiliated with ‘Mental Health Center of Anhui Province, Hefei, Anhui, PR China’. The correct affiliation is listed below:

Vision Research Laboratory, School of Life Science, University of Science and Technology of China, Hefei, Anhui, PR China.

